# A Coumarin–Imidazothiadiazole Derivative, SP11 Abrogates Tumor Growth by Targeting HSP90 and Its Client Proteins

**DOI:** 10.3390/molecules28135226

**Published:** 2023-07-05

**Authors:** Snehal Nirgude, Shahana M. V., Febina Ravindran, Sujeet Kumar, Shivangi Sharma, Raghunandan Mahadeva, Anisha Mhatre, Subhas S. Karki, Bibha Choudhary

**Affiliations:** 1 Institute of Bioinformatics and Applied Biotechnology, Electronic City Phase 1, Bangalore 560100, Karnataka, India; snehalnirgude3490@gmail.com (S.N.); shahanaazad96@yahoo.com (S.M.V.); feina28@gmail.com (F.R.); s_shivangi@ibab.in (S.S.); raghunandan.hunsur@gmail.com (R.M.); anisha.mhatre33@gmail.com (A.M.); 2 Division of Human Genetics, Children’s Hospital of Philadelphia, Philadelphia, PA 19104, USA; 3 Dr. Prabhakar B. Kore Basic Science Research Laboratory Center (Off-Campus), Department of Pharmaceutical Chemistry, KLE College of Pharmacy, Rajajinagar, (A Constituent Unit of KLE Academy of Higher Education; Research, Belagavi), Bangalore 560010, Karnataka, India; klempharma@gmail.com; 4 Department of Biochemistry, Indian Institute of Science, Bangalore 560012, Karnataka, India

**Keywords:** HSP90 inhibitor, DLA mouse tumor model, apoptosis, anticancer therapeutics

## Abstract

Despite several treatment options for blood cancer, mortality remains high due to relapse and the disease’s aggressive nature. Elevated levels of HSP90, a molecular chaperone essential for protein folding, are associated with poor prognosis in leukemia and lymphoma. HSP90 as a target for chemotherapy has been met with limited success due to toxicity and induction of heat shock. This study tested the activity of an HSP90 inhibitor, SP11, against leukemic cells, mouse lymphoma allograft, and xenograft models. SP11 induced cytotoxicity in vitro in leukemic cell lines and induced cell death via apoptosis, with minimal effect on normal cells. SP11 induced cell death by altering the status of HSP90 client proteins both in vitro and in vivo. SP11 reduced the tumor burden in allograft and xenograft mouse models without apparent toxicity. The half-life of SP11 in the plasma was approximately 2 h. SP11 binding was observed at both the N-terminal and C-terminal domains of HSP90. C-terminal binding was more potent than N-terminal binding of HSP90 in silico and in vitro using isothermal calorimetry. SP11 bioavailability and minimal toxicity in vivo make it a potential candidate to be developed as a novel anticancer agent.

## 1. Introduction

Despite advances in cancer therapy, the cancer burden is rising. Resistance to standard-of-care drugs leads to cancer relapse. Cancer is a complex disease, but most cancers share common features that are reviewed as hallmarks of cancer [1,2]. There are several proteins whose loss of function (tumor suppressors)/gain of function (oncogenes) lead to converting a normal cell to a cancer cell. For a protein to function, the folding of a protein is necessary. There are several chaperones in the cellular system which guide protein folding. One such chaperone known to play a role in protein folding in normal cells is HSP90 (Heat shock protein 90) [3,4]. Different isoforms of HSP90 are expressed in cells constitutively and under stress [3,4]. The HSP90 alpha isoform in colorectal carcinoma (CRC) has shown a correlation with advanced tumor stage and shorter recurrence-free survival [5]. HSP90 and co-chaperones modulate protein folding and degradation [3,4]. HSP90 is overexpressed in several cancers, including leukemia, where high expression of HSP90 has been linked to a bad prognosis. HSP90 has chaperone activity for around 400 proteins, some of which are SRC [6,7], AKT [6,7,8], BCL2 [9,10], CDKs [11], etc., which are known to be mutated/activated in cancer cells. HSP90 may stabilize these mutant proteins by preventing degradation. HSP90 is also known to regulate the extracellular matrix (ECM) and has been shown to modulate collagen-I secretion into the ECM [12,13]. Collagen regulates ECM and is known to regulate invasiveness and metastasis [14,15]. Therefore, targeting HSP90 could be one of the strategies to overcome therapy resistance.

**Figure 1:**
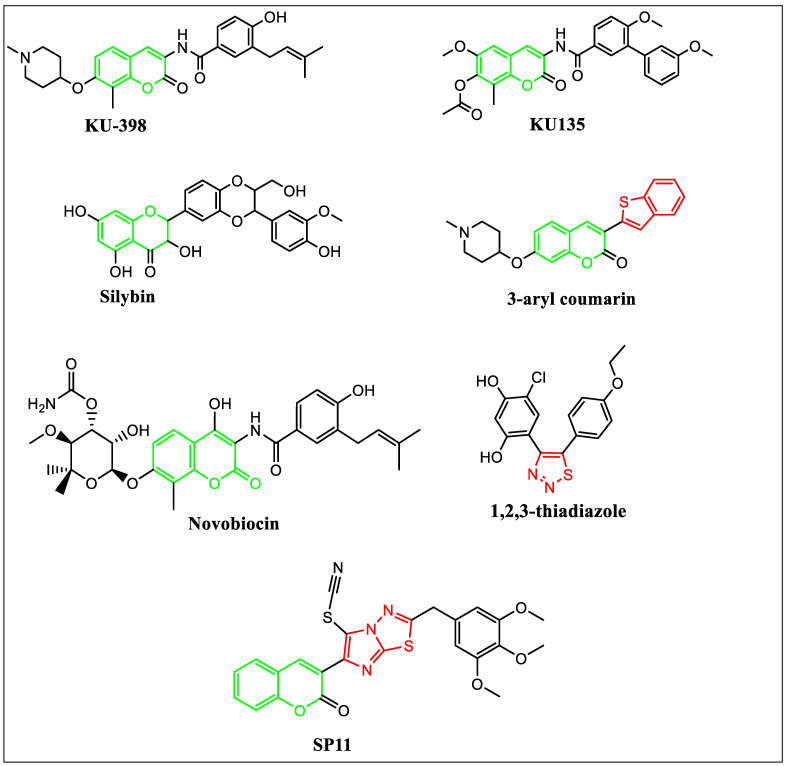
Rationale for the design of SP11.

Several natural and synthetic HSP90 inhibitors targeting the N- and C-terminal domains have been tested for their impact on cell growth. One of the naturally occurring compounds, geldanamycin, and its derivatives 17-AAG and 17-DMAG, have shown cytotoxicity against several cancers [16,17,18,19] but could not make it to the clinic due to toxicity. Novobiocin (4-OH-amino coumarin analog) derived from *Streptomyces niveus*, is another natural antibiotic with HSP90 inhibitory properties, Figure 1 [20,21,22]. The indole-linked 1,3,4-triazole derivative Ganetespib has shown better potency in preclinical studies [23] and has demonstrated inhibitory properties on tyrosine kinase inhibitor (TKI) resistant cancer cells [24,25]. Ganetespib is currently in phase 2 of clinical trials for non-small-cell lung carcinoma (NSCLC) [26].

The activation of HSF1 (heat shock transcription factor 1) is the main limitation of N-terminal domain inhibitors leading to the activation of prosurvival factors in cancerous cells [27]. Therefore, C-terminal domain inhibitors for HSP90 were developed and investigated. Recently, an amino coumarin derivative KU-398 has displayed promising anticancer activity by inhibiting the C-terminal domain of HSP90 [28]. Silybin (silibinin) is a 4-chromanone flavonoid from silymarin identified as an HSP90 inhibitor [28]. KU135, a novel novobiocin derivative, has been tested as an HSP90 inhibitor [29,30]. Huiping et al. have reported 3-aryl coumarin derivatives as antiproliferative agents by HSP90 inhibition [27]. Cikotiene et al. have reported 4-chloro-6-(5-(4-ethoxyphenyl)-1,2,3-thiadiazol-4-yl)benzene-1,3-diol as inhibitors of the HSP90 chaperone. It showed binding affinity to the full-length human HSP90 protein and the N-terminal domain of human HSP90 [31].

Based on these observations, a novel molecule SP11 was designed by linking the coumarin moiety of KU-398, KU135, novobiocin and silibinin with sulfur-and-nitrogen-containing heterocyclic moiety imidazothiadiazole in the third position (Figure 1).

Coumarin, a benzopyrone, has multiple substitution sites and has shown antitumorigenic, antibacterial, antihypertensive, and antiinflammatory activity [32,33]. The coumarin derivatives have shown activity against the prosurvival signals, PI3k/AKT/mTOR pathway, and multi-drug resistance. Also, thiadiazoles have shown antitumor properties by blocking the activity of HSP90 and thiadiazole-containing compounds are cell membrane permeable [34].

This study aimed at the synthesis and characterization of a novel derivative of coumarin and its impact on cytotoxicity in vitro and in vivo in the mouse DLA (Dalton’s Lymphoma Ascites) xenograft model. Also, the bioavailability and toxicity of the compound SP11 were investigated. We also performed in silico ligand protein docking and drug-interaction studies.

## 2. Results

### 2.1. Synthesis

2-amino-5-(3,4,5-trimethoxybenzyl)-1,3,4-thiadiazole (**3**) was prepared by adding phosphorous oxychloride to a mixture of 3,4,5-trimethoxyphenyl acetic acid (**1**) and thiosemicarbazide (**2**) slowly with stirring at room temperature and later refluxed for 1 h, followed by addition of water and potassium hydroxide for attaining basic pH. 3-(2-(3,4,5-trimethoxybenzyl)imidazo [2,1-*b*][1,3,4]thiadiazol-6-yl)-2*H*-chromen-2-one) (**5**) was prepared by refluxing for 10–12 h equimolar quantity of 2-amino-5-(3,4,5-trimethoxybenzyl)-1,3,4-thiadiazole (**3**) and 3-(2-bromoacetyl)-2*H*-chromen-2-one (**4**) in ethanol and neutralized with an aqueous solution of Na_2_CO_3_. Thiocyanate was introduced by reacting compound 5 with potassium thiocyanate in the presence of bromine in glacial acetic acid.

**Figure 2:**
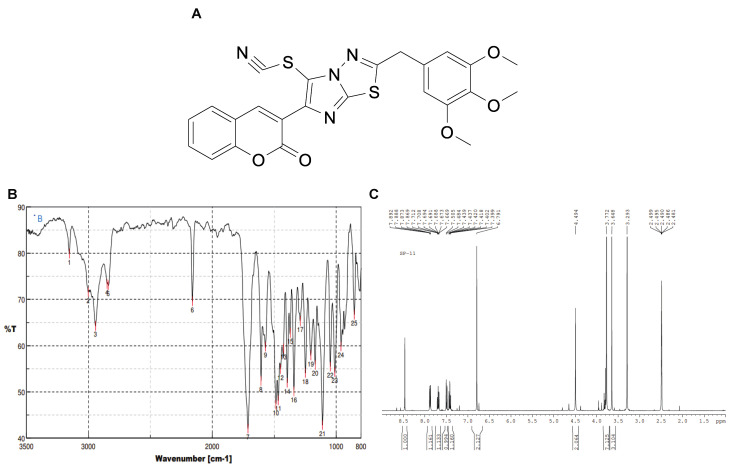

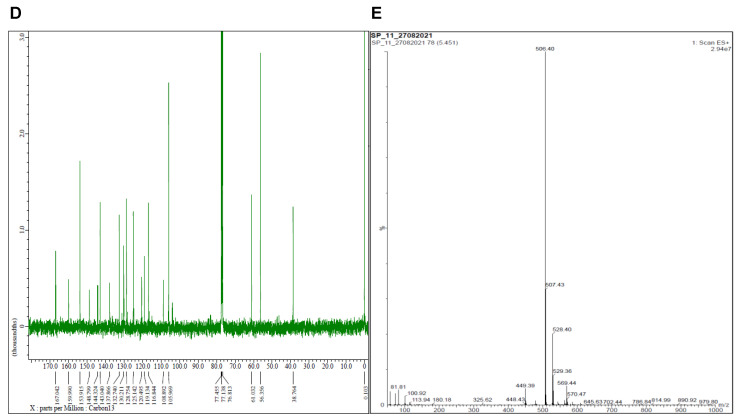
Characterization of SP11. (**A**) Structure of SP11. (**B**) Infrared spectroscopy data of SP11 (%T-Transmittance). (**C**) ^1^HNMR data of SP11. (**D**) ^13^CNMR spectra of SP11. (**E**) LCMS spectra of SP11.

Infrared spectroscopy showed characteristic C-H stretching vibrations in 3178–3001 cm^−1^. Aliphatic C-H stretching vibrations were exhibited at 2940–2834 cm^−1^ and C = O vibrations were observed between 1731 and 1712 cm^−1^. The **SP11** (Figure 2A) showed characteristic vibration at 2161 cm^−1^ for C≡N (Figure 2B).

The intermediate compound, 2-amino-5-(3,4,5-trimethoxybenzyl)-1,3,4-thiadiazole, showed its presence of NH_2_ protons at δ 6.99 ppm as a singlet. The imidazole proton at C_5_ for compound **5** was seen at δ 8.63 ppm in their ^1^H NMR, confirming the cyclization of 2-amino-5-(3,4,5-trimethoxybenzyl)-1,3,4-thiadiazole **3** with 3-(2-bromoacetyl)-2*H*-chromen-2-one **4**. Substitution of thiocyanate (S-C≡N) at the C_5_ position of imidazo [2,1-*b*][1,3,4]thiadiazole has been confirmed by the absence of C_5_-H proton in the ^1^H NMR of compound **6** (**SP11**) (Figure 2C). Aromatic protons of all the compounds (**4**,**5** and **6** (**SP11**)) were shown between δ 8.54 and 6.59 ppm. Protons of –CH_2_- were observed at δ 4.49–4.07 ppm in their ^1^H NMR (Figure 2C). The methoxy protons of the compounds were observed as singlets between δ 3.77 and 3.63 ppm. The ^13^C NMR exhibited peak at δ 167 ppm for C=O, peaks between δ 160–106 ppm for aromatic carbons and peaks at δ 61 and 56 ppm for OCH_3_, and at δ 39 ppm for –CH_2_- (Figure 2D).Finally, compound **6** (**SP11**) was confirmed for their mass by LCMS (Figure 2E). The purity of the compound was ascertained by HPLC and found to be 88%.

### 2.2. SP11 Induces Cytotoxicity in Various Leukemia Cell Lines with Least Effect on Normal Cells

**Figure 3:**
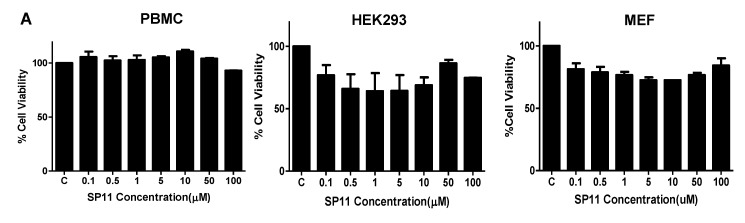

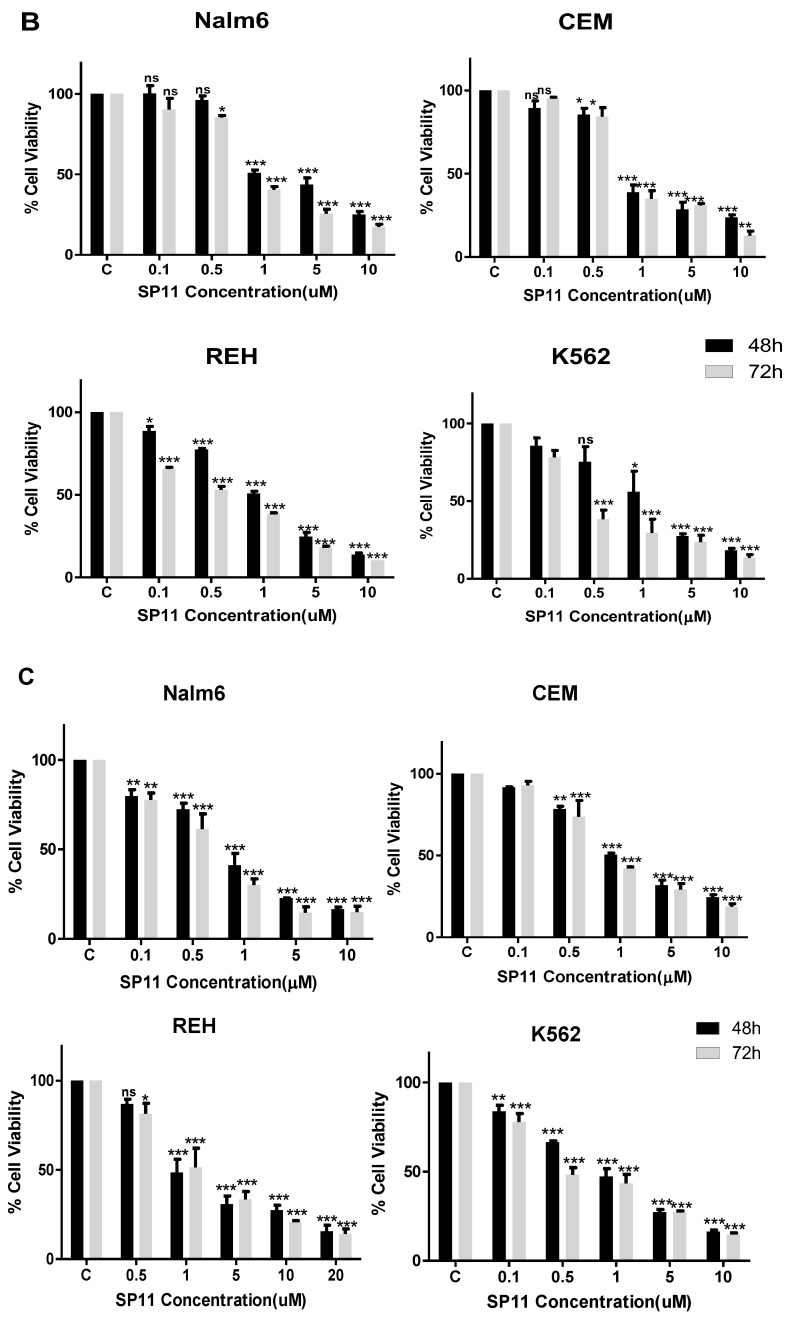
Evaluation of cell viability after SP11 treatment: (**A**) Bar graph depicting cell viability upon SP11 treatment of normal cell lines as tested by MTT assay. (**B**) Bar graph depicting cell viability upon SP11 treatment of leukemic cell lines as tested by MTT assay. (**C**) Bar graph depicting cell viability upon SP11 treatment of leukemic cell lines as tested by LDH assay. Data from 3 different biological replicates were collected for all experiments and were presented as bar graphs using Graph Pad prism tool. Two-way ANOVA followed by Tukey’s multiple comparison test was carried out and significance is represented as *** *p*-value ≤ 0.001, ** *p*-value ≤ 0.01, * *p*-value ≤ 0.05, ns = not significant.

We studied the cytotoxic effect caused by SP11 on five different leukemic cell lines (Table 1)—Nalm6 (B-cell leukemia) [35], CEM (T-cell leukemia) [36], K562 (chronic myelogenous leukemia) [37], REH (B-cell leukemia) [38], and Molt4 (human T-lymphoid) [39]. We also assessed cytotoxicity of SP11 on normal cells—HEK293 (normal kidney cell line), MEFs (normal fibroblast cell line), and PBMC (peripheral blood mononuclear cells) (Figure 3A). To evaluate the effect of SP11 on cell proliferation, LDH and MTT assays were performed. MTT (Figure 3B) and LDH assay (Figure 3C, Supplementary Figure S1) showed that SP11 could efficiently inhibit the growth of leukemic cell lines Molt4, Nalm6, followed by CEM, REH and K562 cells. IC_50_ was estimated to be 0.72, 0.847, 0.889, 1.06, and 1.26 µM, respectively, for these cell lines (at 48 h). We observed minimal cytotoxicity towards normal cells—293, MEFs and PBMCs at the highest concentration of 100 µM. Since Molt4 and Nalm6 cells exhibited maximum sensitivity towards SP11, they were selected for subsequent in vitro and in vivo studies.

### 2.3. SP11 Treatment Induces Apoptosis Rather Than Necrosis in Leukemic Cells

**Figure 4:**
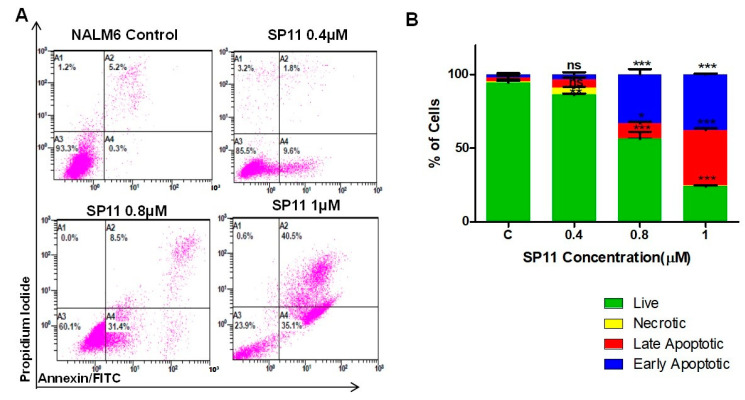

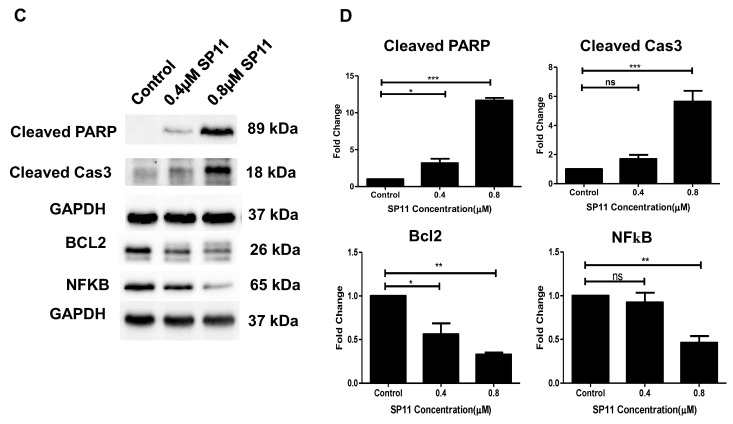
Assessment mode of cell death on SP11-treated Nalm6 cells: (**A**) Dot plot depicting SP11-treated (0, 0.4, 0.8, 1 µM) Nalm6 cells for 48 h and then stained with annexin-FITC/PI. (**B**) Quantification of Nalm6 cells at each stage. Every experiment was repeated thrice and two-way ANOVA test was performed. *p*-value was calculated between control and SP11-treated groups (*** *p*-value ≤ 0.001, ** *p*-value ≤ 0.01, * *p*-value ≤ 0.05, ns = not significant). (**C**) Western blot analysis of apoptotic markers was performed on SP11 (0, 0.4, 0.8, 1 µM for 48 h) treated Nalm6 cell lysates. Each experiment was performed in duplicate and representative image is shown for each marker. (**D**) Quantification was performed for each marker and is represented as bar graph of mean ± SEM. One-sample *t* test and one-way ANOVA test were performed, and the *p* value was calculated between control and ST09-treated groups (*** *p*-value ≤ 0.001, ** *p*-value ≤ 0.01, * *p*-value ≤ 0.05, ns = not significant).

To decipher the SP11-induced cell death mechanism (apoptosis/necrosis) in Nalm6 cells, annexin-FITC/PI staining was performed. Annexin V recognizes the phosphatidylserine that flips towards the outer side of the plasma membrane in cells undergoing apoptosis [40]. To distinguish necrotic cells from apoptotic cells, the cells were costained with propidium iodide (PI), excluded by apoptotic cells. SP11-treated (0, 0.4, 0.8, and 1 µM, 48 h) Nalm6 cells showed positive staining for annexin-FITC and annexin-FITC/PI, suggesting the presence of both early and late apoptosis upon treatment (Figure 4A,B). These results indicated induction of apoptosis rather than necrosis in SP11-treated Nalm6 cells.

Alteration in the expression of apoptotic markers like cleaved PARP, cleaved caspase 3 and Bcl2 were also assayed in SP11-treated (0, 0.4, 0.8 µM) Nalm6 cells after 48 h of treatment (Figure 4C,D). The concentration-dependent increase was observed in protein levels of cleaved PARP and caspase 3. In contrast, a significant decrease was observed in Bcl2 protein in a concentration-dependent manner. These results indicate that SP11 induced cytotoxicity in Nalm6 cells via apoptosis. We also performed in-cell Western on the MOLT-4 cell line for Bax, caspase-9, and Bcl2 proteins. BAX and cleaved caspase-9 were elevated on SP11 treatment in MOLT-4 cell lines as shown in the Supplementary Figure S2A–D while BCL2 expression was found to be reduced with SP11 treatment.

Since SP11 showed promising anticancer properties in vitro in leukemia cells, in vivo evaluation of tumor regression was performed on the DLA mouse model. DLA is a well-characterized, reproducible, and transplantable mouse tumor model [41].

### 2.4. SP11 Induces Tumor Regression in DLA Induced Allograft Mouse Tumor Model

**Figure 5:**
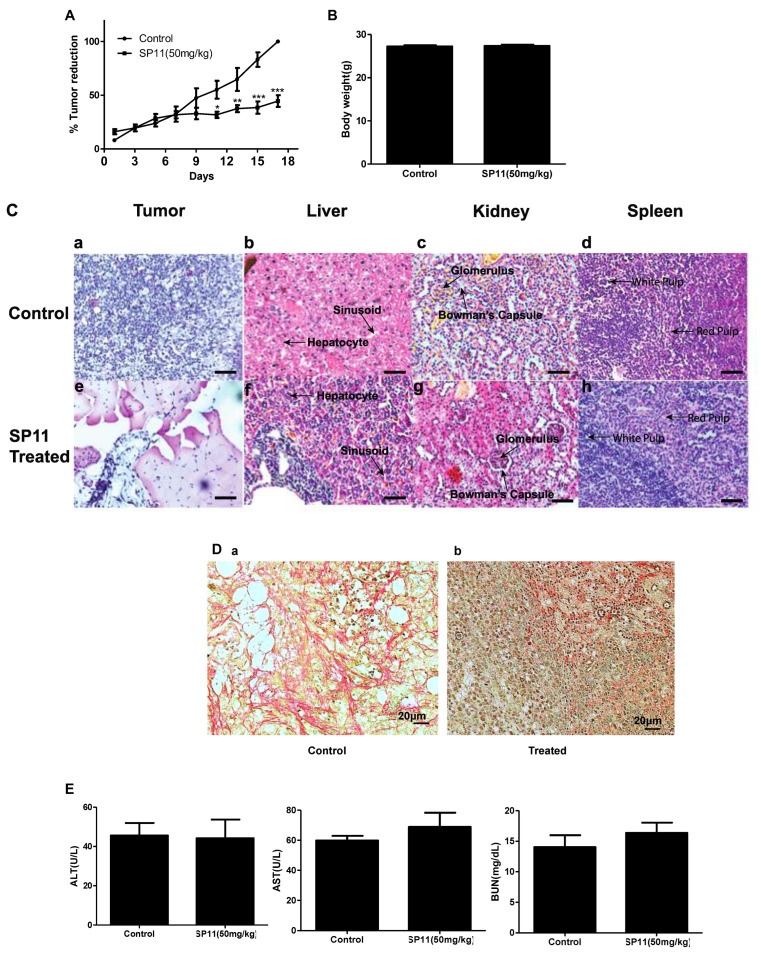

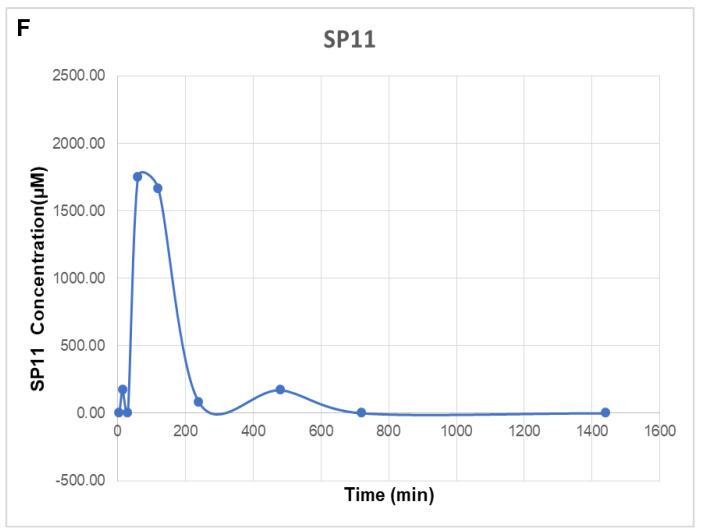
Evaluation of effect of SP11 on tumor growth, its toxicity tests, and bioavailability study: DLA cells (1 × 10^6^ cells/animal) were injected to induce solid tumors. After the 7th day of injection, i.p. injection with SP11 (50 mg/kg b.wt) was started every alternate day throughout the experiment period. (**A**) Tumor volume was calculated before SP11 treatment. (**B**) Body weight of animals at the end of the study. (**C**) Histopathological analysis of tumor and organs after SP11 treatment. At the end of the study, tumor tissue and organs were collected and used for histological analysis. Representative images of H&E-stained sections of (**a**). control tumor, (**e**). SP11 post-treatment tumor, (**b**). control liver, (**f**). post-treatment liver, (**c**). control kidney, (**g**). ST09 post-treatment kidney, (**d**). control spleen, (**h**). post-treatment spleen (scale bar = 20 μM). (**D**) Collagen staining. (**a**) Collagen (picrosirius) stained paraffin sections of control tumor, (**b**) SP11 treatment tumor. (**E**) Blood ALT, AST, urease test results plotted as bar graph. Blood was collected at the end of the study. (**F**) Bioavailability study profile of SP11. Plasma concentration–time profile of intraperitoneal administration of SP11 was determined by HPLC-UV. SP11 (50 mg/kg bodyweight) was administered intraperitoneally into Swiss albino mice. Plasma concentrations are represented in each graph along Cmax and Tmax of the drug. Each point represents the mean concentration (n = 2).

Tumor was developed in Swiss albino mice, by injecting DLA cells, following which animals were treated with SP11 (50 mg/kg bd wt, 9 doses), and the rate of growth of tumor was monitored over a period of 17 days. SP11 treatment resulted in significant tumor burden reduction compared to the control group (Figure 5A). No significant weight reduction was observed in either group (Figure 5B). The tumor, liver, spleen, and kidney sections of mice from both (control and treatment) groups were analyzed for changes in the morphology post SP11 treatment. The H&E staining showed no significant changes in cellular morphology in kidney, liver, and spleen tissues of either group (Figure 5C). This indicates that SP11 induces no significant adverse effect on vital organs. The H&E-stained tumor tissue from the untreated control group showed densely stained and packed nuclei, indicating more proliferating cells compared to the SP11-treated tumor section. Picrosirius red staining was performed to study the effect of SP11 on collagen, a major component of the extracellular matrix. The staining showed a significant reduction in collagen in the post-SP11-treated tumor when compared to the control tumor (Figure 5D).

### 2.5. SP11 Reduced Tumor Burden with Minimal Toxicity and Enhanced Bioavailability

To check whether SP11 treatment led to systemic toxicity, serum was collected for analysis from the blood of all animals in study. Liver and kidney functions were assessed by markers such as aspartate aminotransferase (AST), alanine aminotransferase (ALT), and urea. AST, ALT, and BUN were in the normal range for both the groups in the study (AST < 100 U/L and ALT < 60 U/L). This indicated that SP11 induced no adverse effects in liver and kidney functions (Figure 5E).

Bioavailability studies were performed using the plasma of a single dose of SP11 50 mg/kg body weight for female Swiss albino mice. After the intraperitoneal plasma collection at different time points (5, 10, and 15 min, and 1, 3, 6, 12, and 24 h), it was analyzed using the Shimadzu LC-20A system. The system was equipped with the UV–visible spectrometry detector SPD-M20A photodiode array. The maximum concentration (Cmax) of SP11 was 1750.89 μM at 1 h intraperitoneally and t1/2 was 113 min (Figure 5F).

### 2.6. SP11 Binds to the C-Terminal of HSP90

**Figure 6:**
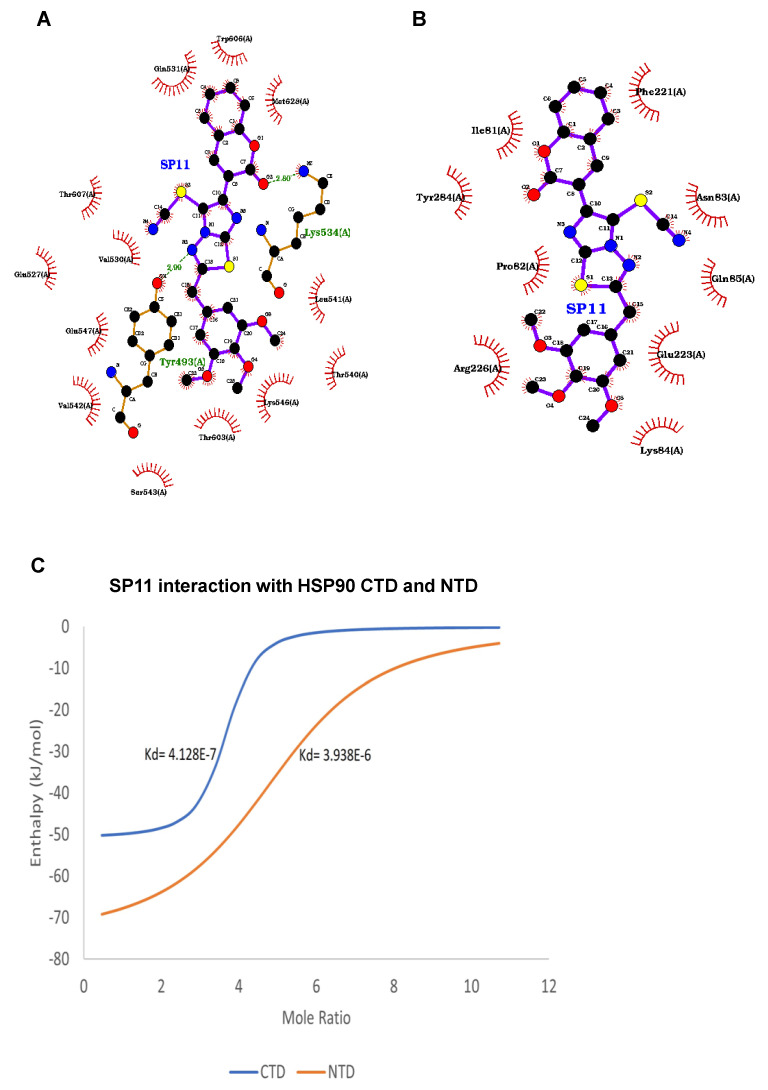

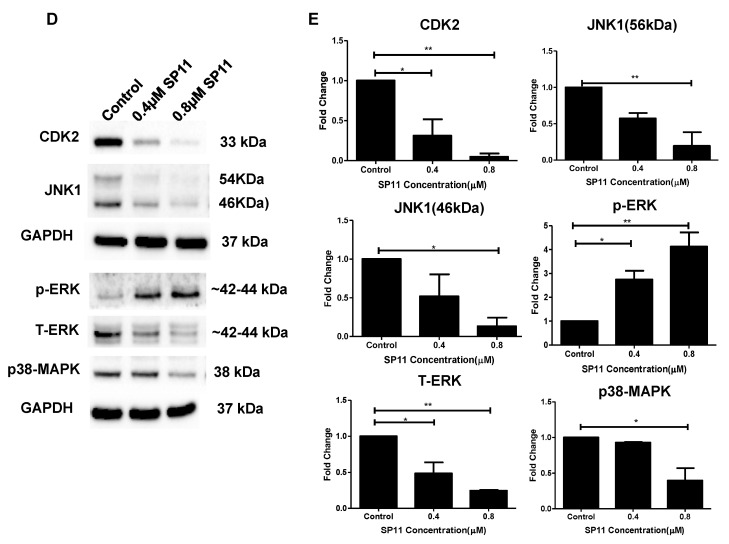
Ligand interaction map of SP11 and assessment HSP90 client proteins on SP11-treated Nalm6 cells: Ligand interaction map of SP11 with (**A**) HSP90 C-terminal domain and (**B**) HSP90 N-terminal domain bound in the ATP-binding pocket of human Hsp90 visualized using LigPLot+. Hydrogen bonds are shown as green dotted lines, while the spoked arcs represent residues making nonbonded contacts with the ligand. (**C**) Binding isotherm for HSP90 CTD and NTD against SP11. (**D**) Assessment of HSP90 client proteins in SP11 (0, 0.4, 0.8, and 1 µM for 48 h) treated Nalm6 cell lysates. Each experiment was performed in duplicate and representative image is shown for each marker. (**E**) Quantification was performed for each marker and is represented as bar graph of mean ± SEM. One-sample *t* test and one-way ANOVA test were performed, and the *p* value was calculated between control and SP11-treated groups (*: *p* value ≤ 0.05, **: *p* value ≤ 0.005).

After observing promising anticancer effects, we performed molecular docking studies to check the binding of SP11 to HSP90. Autodock vina was used to dock the ligand SP11 with the HSP90 molecule and LigPlot+ software for visualizing the protein ligand interactions (Figure 6A,B). At the C-terminal, residues Lys534 and Tyr493 engaged in direct hydrogen bond interactions at distances 2.88 A and 2.99 A, respectively. Residues Gln531, Trp 606, Met628, Leu 541, The 540, Lys 546, Thr 603, Val542, Glu547, Val530, Glu 527, and Thr607 interacted hydrophobically with the SP11 ligand together resulting in an affinity of −8.5 kcal/mol which was observed to be highest for SP11 with HSP90 (Figure 6A). Conversely, at the N-terminal domain only hydrophobic interactions were observed with Phe221, Asn83, Glu85, Glu223, Lys84, Arg226, Pro 82, Tyr284, and Ile81, implying that SP11 binds better at the C-terminal domain (Figure 6B).

### 2.7. Isothermal Calorimetry

In silico studies showed promising results and hence we performed the drug–protein interaction studies using an isothermal calorimetry approach. First, we isolated and purified the CTD and NTD of HSP90 protein (Supplementary Figure S3A,B). Nano-ITC results for SP11 with both CTD and NTD showed heat release (negative enthalpy value) indicating drug–protein interaction (Supplementary Figure S4). The Kd values of SP11-HSP90 CTD and SP11-HSP90 NTD are 4.128 × 10^−7^ and 3.829 × 10^−6^ respectively (Figure 6C). The low dissociation constant for CTD compared to NTD implies the higher binding affinity of SP11 towards CTD compared to NTD. The results were analyzed using Launch NanoAnalyze software from TA Instruments.

### 2.8. SP11 Regulates Expression of HSP90 Client Proteins

After confirming SP11-HSP90 interaction studies, we checked the expression profiles of five HSP90 client proteins—CDK2 [42], JNK1 [43], p-ERK [6,44,45], T-ERK [6,43,46], and p38-MAPK [46]. SP11 downregulated CDK2, JNK1, T-ERK, and p38-MAPK protein levels. p-ERK levels were upregulated upon SP11 treatment (Figure 6D,E). This shows that SP11 interacts with SP11 and regulates expression of its client proteins.

### 2.9. SP11 Induces Tumor Regression in a Xenograft Mouse Model and Regulates Expression of HSP90 Client Proteins

**Figure 7:**
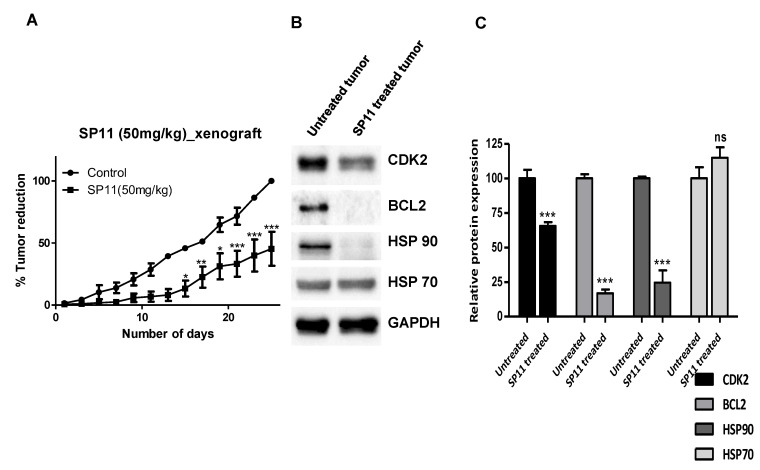
SP11-mediated tumor regression in xenograft mouse tumor model: Molt4 (5 × 10^6^ cells/animal) were injected into nude mice to induce solid tumors. Oral administration of SP11 (50 mg/kg bd. wt) daily was carried out for the duration of the experiment. (**A**) Tumor volume profile after SP11 treatment. (**B**) Effect of SP11 on the expression of HSP90 client proteins in the tumor tissue of experimental animals. Tissue lysates were prepared from the dissected tumor samples of post treatments. An amount of 40 µg of protein was loaded in SDS-PAGE and checked for protein expression by Western blotting. (**C**) Quantification was performed for each marker and is represented as a bar graph of mean ± SEM. One-sample *t*-test was performed, and the *p*-value was calculated between control and SP11-treated groups (*** *p*-value ≤ 0.001, ** *p*-value ≤ 0.01, * *p*-value ≤ 0.05, ns = not significant).

To evaluate the potential of SP11 to be used as a chemotherapeutic agent, we further analyzed its effect on tumor regression in vivo using a xenograft model. We developed a xenograft model by implanting Molt4 cells in nude mice. A significant reduction in the tumor volume of SP11-treated mice (50 mg/kg, 13 doses) was observed by the 26th day of treatment (Figure 7A). Thus, our data suggest that both the tumor models allograft and xenograft tested exhibited remarkable sensitivity to SP11 administration.

We further assessed the expression of HSP90 protein and its client proteins in a Molt4 xenograft tumor. As compared to control, the SP11-treated tumors had significant reduction in HSP90 and client proteins like CDK2 and Bcl2(Figure 7B,C). HSP70 levels were unaffected. This shows that HSP90 is the major target of SP11 and does not induce heat shock response, while there was reduction in the levels of client proteins which would signal cell proliferation.

## 3. Discussion

The present study elucidated the cell death mechanism induced by SP11, a novel synthetic compound. SP11 exhibited promising antitumorigenic properties in leukemic cells in vitro and lymphoma (DLA, xenograft) models in vivo. Importantly, we observed that SP11 reduced tumor burden significantly with minimal toxicity. In addition, SP11 targets HSP90 and regulates its client proteins to execute its antiproliferative properties.

Despite numerous efforts to cure leukemia, relapse is observed in a large fraction within 2–5 years [47]. Among lymphomas, non-Hodgkin lymphoma (NHL) has the worst prognosis. Several HSP90 inhibitors have been designed to competitively bind to the N-terminal domain of HSP90 and have also entered clinical trials [48,49,50]. Only 18% individuals in the phase I clinical trial had remission in the phase I trial using KOS-1022, an N-terminal HSP90 inhibitor [51]. Zong et al., 2015 described activation of the signalosome in acute myeloid leukemia (AML) cells which is driven by the presence of tumor-specific HSP90 [52]. Different cells and patients might activate different signals and, depending on the number of signals activated and levels of HSP90, drugs against HSP90 may be beneficial. In chronic myelogenous leukemia (CML), HSP90 is known to protect the fusion protein BCR-ABL maintaining kinase activity [53]. Similarly in diffuse large B-cell lymphoma (DLBCL), HSP90 complex with BCL6 leads to gene silencing by binding to the promoter region [54].

One of the major drawbacks of the N-terminal domain inhibitor of HSP90 was the induction of heat shock response [55,56,57] and this may be the reason for failure in clinical trials. Further, HSP90 inhibitors targeting the C-terminal domain [58] have shown promising results and do not lead to induction of heat shock response.

The molecule SP11 was designed by keeping the coumarin ring present in KU-398, KU135 and from silybin, and substitution was made at the third position by imidazothiadiazole. KU-398 and KU135 as well as silybin have been shown to bind to the C-terminal domain of HSP90 and modulate cytotoxicity in breast cancer cells and leukemic cells by altering HSP90 client proteins [27,29]. We modeled SP11 binding to dimerized HSP90 and found it to bind strongly to the C-terminal domain. We confirmed the drug–ligand interaction by isothermal calorimetry, a gold standard method for drug–ligand interaction study [59].

To study the selectivity of SP11 towards cancer cells over normal cells, we assayed cytotoxicity of normal cells of 293, MEFs and PBMCs as described [60,61]. It was observed that SP11 is at least nine times more cytotoxic towards cancer cells than normal cells. SP11 has the highest IC_50_ value of 1.62 µM towards K562 cells whereas it was non-toxic at 100 µM towards all three normal cells (HEK293, MEFs, and PBMCs) in the study. This highlights the importance of the selectivity of SP11 on cancer cells.

Cancer cells evade cell death leading to their malignant transformation [62,63]. Additionally, reduced apoptosis or resistance has a vital role in tumorigenesis [62]. Hence, there is a demand for compounds that can induce or trigger apoptosis in malignant, aggressive cancer cells leading to their effective elimination. SP11 induced apoptosis in leukemic cells of Nalm6 in the micromolar concentration range. Cleavage of PARP-1 and caspase 3 are known apoptotic markers. Caspase 3 and 7 cleave the 116 kDa form of PARP to generate the 89 kDa fragment [64]. SP11 induced cleavage of both PARP and caspase 3, leading to apoptosis. Bcl2 is a pro-survival gene that inhibits apoptosis [65,66]. Hence, its downregulation in cancer is of vital importance. SP11 significantly downregulated Bcl2 in leukemic cells, a client protein stabilized by HSP90.

HSP90 and co-chaperones regulate protein folding and degradation of client proteins. In our study, CDK2, JNK1, and p38MAPK, and downstream signaling molecule NF-kB were downregulated, and p-ERK1/2 was upregulated upon SP11 treatment. HSP90 client proteins CDK2 [42], JNK1 [43], and p38-alpha MAPK are elevated in leukemia (GEPIA) and are clients of HSP90 [67]. Downregulation of the kinases mentioned above led to apoptosis as observed in SP11-treated cells, except for ERK, which was upregulated. In that context, ERK has a dual role and has been shown to have a proapoptotic role [68]. SP11 also targeted HSP90 and its client proteins in the humanized mouse model of leukemia. Along with significant tumor reduction, SP11 reduced expression of Bcl2, HSP90, and CDK2 proteins. SP11 selectively reduced HSP90 protein levels when compared to HSP70, highlighting its specificity of drug mechanism.

Collagen production by cancer cells and TAMs are therapeutic targets. Almost all cancer types have elevated levels of collagen (GEPIA). Collagen can transduce signals via integrins, discoidin domain receptors, and tyrosine kinase receptors, and influence tumor cell growth and behavior. Wong et al. described the role of HSP90 in collagen 1 secretion and its inhibition by HSP90 inhibitors [69]. SP11 inhibited collagen as revealed by picrosirius red staining of the treated tumor. SP11 can also function by modulating collagen production.

Cell proliferation refers to how a cell can quickly divide itself, which is a hallmark of cancer cells [70]. H&E staining showed densely stained and packed nuclei in the untreated control tissue section compared to the treated tissue section. This indicated that SP11 mainly targeted proliferating cells. While targeting cell proliferation in carcinogenesis is essential, many cytotoxicity-inducing drugs also target rapidly proliferating, non-tumor cells in the body [71,72]. This leads to increased toxicity induced by the drug. Most of the HSP90 inhibitors tested could not get clinical approval due to toxicity and in vivo stability [73,74,75]. Several HSP90 inhibitors reviewed by Li et al. reached phase I clinical trials but dropped from trials due to toxicity, and several are in clinical trials due to the potential that HSP90 targeting offers in terms of regulating the stability of the oncoproteins and cell survival signaling [3]. SP11 led to tumor regression with the least drug toxicity while maintaining the functions of vital organs like the liver and kidney. The lack of apparent toxicity could be due to its short half-life of around 2 h and the concentration maxima of roughly 60% of the administered drug. One of the drawbacks of this analysis is the absence of measurement of the drug in tumors, which is the only way to measure HSP90 activity as the bioavailability in the blood did not correlate with HSP90 inhibitors [76].

The in vitro and in vivo experiments revealed SP11 interacts with HSP90 at the C-terminal and regulates client proteins’ stability and regulates apoptosis by activating the proapoptotic proteins and reducing antiapoptotic proteins. SP11 was bioavailable and did not show any apparent renal or hepatotoxicity. SP11, a coumarin and thiadiazole integrated anticancer drug, has the potential to be developed as an anticancer drug.

## 4. Material and Methods

### 4.1. Synthesis of 3-(2-(3,4,5-Trimethoxybenzyl)imidazo [2,1-b][1,3,4]thiadiazol-6-yl)-2H-chromen-2-one) (***5***)

**Figure 8:**
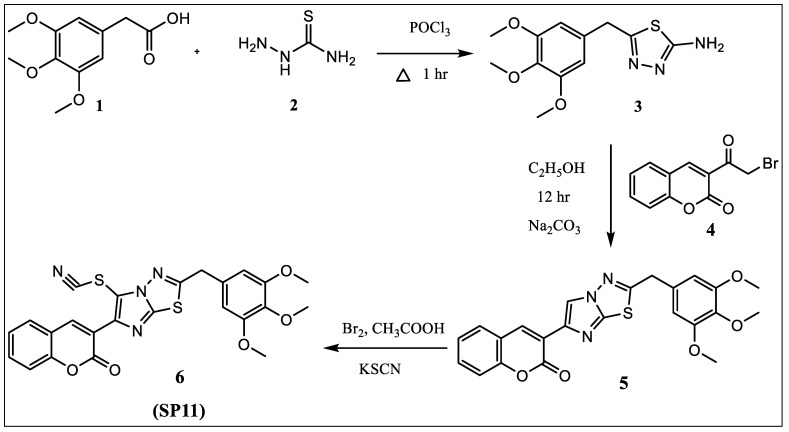
Synthesis scheme of SP11.

Equimolar quantity of 2-amino-5-(3,4,5-trimethoxybenzyl)-1,3,4-thiadiazole (**3**) and 3-(2-bromoacetyl)-2*H*-chromen-2-one (**4**) in ethanol was refluxed for 10–12 h (Figure 8). The reaction mixture was poured in ice-cold water and pH of the solution was adjusted to 7.0 with aqueous solution of Na_2_CO_3_ to get 3-(2-(3,4,5-trimethoxybenzylimidazo [2,1-*b*][1,3,4]thiadiazol-6-yl)-2*H*-chromen-2-one (**5**). The compound so obtained was purified from chloroform–ethanol mixture.

White amorphous powder, yield: 53%, MP; 209–211 °C, IR (*v* cm^−1^): 3178, 2940, 2834, 1731, 1597, 1510, 1471, 1338, 1131. ^1^HNMR (400 MHz, DMSOd_6_) δ ppm: 8.63 (S, 1H, Imi-H), 8.54 (s, 1H, Coum-H), 7.83 (d, 1H, *J* = 9.2), 7.57 (t, 1H, *J* = 17.6), 7.42 (d, 1H, *J* = 8.4), 7.35 (t, 1H, *J* = 15.2), 6.71 (s, 2H, ar), 4.35 (s, 2H, -CH_2_-), 3.73 (s, 6H, 2-OCH_3_), 3.61 (s, 3H, -OCCH_3_). ^13^C NMR (100 MHz, CDCl_3_) δ ppm: 164.37 (s), 159.60 (s), 153.79 (s), 152.80 (s), 146.20 (s), 138.73 (s), 137.60 (s), 136.37 (s), 131.19 (s), 130.63 (s), 128.16 (s), 124.73 (s), 119.80 (s), 119.70 (s), 116.48 (s), 115.01 (s), 105.98 (s), 61.02 (s), 56.30 (s), 38.70(s).

### 4.2. Synthesis of 3-(5-Thiocyanato-2-(3,4,5-trimethoxybenzyl)imidazo [2,1-b][1,3,4]thiadiazol-6-yl-2H-chromen-2-one ***6*** (***SP11***)

An amount of 0.0025 M of bromine in glacial acetic acid (10 mL) was added dropwise at 0 °C to a solution of 0.0025 M of 3-(2-(3,4,5-trimethoxybenzyl)imidazo [2,1-*b*][1,3,4]thiadiazol-6-yl)-2*H*-chromen-2-one (**5**) and 0.004 M of potassium thiocyanate in 10 mL of glacial acetic acid (Figure 8). The reaction mixture was further stirred for 1 h at 15–18 °C, after which it was poured into ice-cold water. The solid that separated was filtered and recrystallized from the mixture of chloroform/ethanol.

White amorphous powder, yield: 55%, MP; 205–209 °C, IR (*v* cm^−1^): 3154, 3001, 2943, 2850, 2161, 1712, 1607, 1487, 1341. ^1^HNMR (400 MHz, DMSOd_6_) δ ppm: 8.47 (s, 1H), 7.89 (d, 1H, *J* = 9.2), 7.69 (t, 1H, *J* = 17.2), 7.51 (d, 1H, *J* = 8.4), 7.42 (t, 1H, *J* = 16), 6.79 (s, 2H), 4.49 (s, 2H, -CH_2_-), 3.77 (s, 6H, 2-OCH_3_), 3.65 (s, 3H, -OCH_3_). ^13^C NMR (100 MHz, CDCl_3_) δ ppm: 167.04 (s), 160.00 (s), 153.92 (s), 153.91 (s), 148.80 (s), 144.32 (s), 143.04 (s), 137.87 (s), 132.74(s), 130.21 (s), 128.75 (s), 125.14 (s), 124.60 (s), 120.49, (s), 119.13 (s), 116.84 (s), 116.40 (s), 108.89 (s), 105.97 (s), 104.00 (s), 61.03 (s), 56.36 (s), 38.76 (s). LCMS (ESI) *m*/*z*: 506.40 (506.55).

### 4.3. Reagents, Chemicals, and Apparatus

The IR spectra were recorded in KBr on a Jasco 430+; the ^1^H NMR spectra were recorded in CDCl_3_/DMSO on a Bruker (400 or 100 MHz) and J values were reported in hertz (Hz) for ^1^H NMR. 3-(2-bromoacetyl)-2*H*-chromen-2-one was prepared as per the literature [77]. The LCMS spectrum of SP11 was recorded on triple-quadrupole liquid chromatography mass spectrometry (LC-MS) 6410 from Agilent Technologies.

Ponceau S (Sigma life Sciences, Burlington, MA, USA), Phenylmethanesulfonyl chloride (PMSF), protease inhibitor cocktail tablets (EDTA-free), Tris base, Glycine, acrylamide, and bis-acrylamide powder, DMSO, MTT reagent and all other routine chemicals were purchased from MP biomedicals, (Santa Ana, CA, USA).

### 4.4. Cell Culture

HEK293, CEM, K562, Reh, and Molt4 cells were purchased from the National Centre of Cell Culture (NCCS), Pune. CEM, K562, Molt4, and Reh cells were maintained in Roswell Park Memorial Institute-1640 (RPMI; Lonza, Basel, Switzerland) media. Human embryonic kidney (HEK293) and mouse embryonic fibroblast (MEF) cells were maintained in DMEM media (Lonza). All the media were supplemented with heat-inactivated 10% fetal bovine serum (Gibco, New York, NY, USA), 100 IU mg/mL penicillin/streptomycin (Gibco) at 37 °C in a humidified atmosphere containing 5% CO_2_. SP11 was dissolved in DMSO and all treatments were adjusted to equal concentrations of DMSO between 0.1–0.2%. The cytotoxicity of SP11 was assessed on normal cells using HEK293, MEFs, and peripheral blood mononuclear cells (PBMCs). Nalm6 and PBMC cells were a kind gift from SCR lab, IISc, Bangalore, India.

### 4.5. MTT Assay

MTT assay was performed as described earlier [60,61,71]. Briefly, cells (5000 cells/well) were seeded in triplicates in 96-well plates and treated with various concentrations of SP11 after a 24 h incubation. Cells were then treated with MTT reagent, MP Biomedicals (5 mg/mL) at 37 °C, 5% CO_2_, post 48 and 72 h of incubation with SP11. Absorbance was recorded at 570 nm and the results shown were collected from three different biological replicates.

### 4.6. LDH Assay

As described [60,61], a lactate dehydrogenase (LDH) release assay was performed on cells. Briefly, 5000 cells were seeded in each well of the 96-well plate in triplicates. After 24 h incubation, cells were treated with varying concentrations of SP11. After 48 and 72 h of treatment, the absorbance of the orange-red colored formazan product was recorded at 490 nm and data from three different biological replicates were collected and plotted.

### 4.7. Apoptosis Assay

Nalm6 cells were grown in 6-well plates with a density of 80,000/mL and assay was performed as described [59,60,70]. Then the cells were treated with different doses of SP11 (0,0.4 μM, 0.8 μM, 1 μM) for 48 h. Cells were trypsinized, washed with ice-cold 1X Phosphate buffer saline and resuspended in 1X annexin binding buffer containing annexin V-FITC antibody (Biolegend, San Diego, CA, USA) for 15 min in the dark on ice. PI (propidium iodide) was added (3.3 μg/mL) just before acquiring the samples. In total, 10,000 events were acquired for each sample using Beckman coulter Gallios flow cytometer (Beckman Coulter, Miami, FL, USA).

### 4.8. Immunoblotting

The assay was performed as described earlier [60,71,78]. Nalm6 cells (80,000 cells/mL) were seeded and treated with SP11 (0, 0.4, 0.8 μM) for 48 h and the whole-cell lysate was prepared as described [60,61].

### 4.9. In-Cell Western

Briefly, 50,000 cells per well of MOLT-4 cells were plated in a 24-well plate and treated with different concentrations of SP11 for 48 h. Post-incubation cells were fixed in 4% paraformaldehyde, permeabilized with Triton-x in PBST and blocked with intercept blocking buffer (LI-COR, Lincoln, NE, USA) for 20 min. Primary antibodies GAPDH (1:500, Elabscience, Bethesda, MD, USA), BAX (1:200, Cloud- Clone Corp, Houston, TX, USA), BCL2 (1:200, Cloud-Clone Corp), and Cleaved caspase-9 (1:200, Cell signaling technology, Danvers, MA, USA) were added and incubated overnight at 4 °C. Post incubation the cell suspensions were washed 5 times in PBST and secondary anti-rabbit (IRDye^r^ 680RD Goat anti-Rabbit, 925-68071) and anti-mouse (IRDye^r^ 800CW Goat anti-Mouse, 925-32210) were added and incubated for 2 h with them. Results were analyzed using Empiria Studio 2.2.

### 4.10. Dalton’s Lymphoma Tumor Model

The study was approved by the “committee for control and supervision of experiments on animals” (CPCSEA, Government of India, Animal welfare division, Reg. No. 1994/GO/ReBi/S/17/CPCSEA). All experiments were performed following institutional and national guidelines and regulations of the CPCSEA. Dalton’s Lymphoma Ascites (DLA) cells (1 × 10^6^ cells/animal) were injected to induce solid tumors in the left thigh region of female Swiss albino mice. After animals had developed tumors of size ~200 mm^3^, the animals were segregated into 2 groups: control (n = 5) and SP11-treated (n = 5) as described in [60,71]. The treated group animals were then subjected to 9 doses of 50 mg/kg of body weight of SP11 intraperitoneally (i.p.) every alternate day. The experiment was repeated three times with 5 animals each per group to a total number of n = 15. Changes in the tumor size and body weight were observed for 17 days from the day of treatment. Tumor volume was calculated using the formula V = 0.5 × a × b^2^, where V is tumor volume, and a, b are significant and minor tumor diameters.

### 4.11. Drug Toxicity and Side-Effect Assessment on ST09 Treatment

DLA tumor-induced mice in the treatment group were treated with SP11 for 17 days and then were evaluated for drug toxicity using standard enzymatic assays like aspartate aminotransferase (AST), alanine aminotransferase (ALT), and blood urea nitrogen (BUN) as described [60,71].

### 4.12. Histological Analysis of Tumor Tissues

Histological evaluation through hematoxylin–eosin (HE) staining was performed by fixing tumor tissues and organs from animals following treatment with SP11 (50 mg/kg) in formalin. The tissues were then embedded in paraffin and processed as previously described [60,71].

### 4.13. Collagen Staining

Paraffin sections of control and SP11-treated tumors were dewaxed and hydrated, followed by hematoxylin staining. Picrosirius red dye was used for staining collagen [79,80]. Staining with picrosirius red was performed for 1 h, followed by washes in two changes of acidified water and dehydration in three changes of 100% ethanol. Finally, after clearing in xylene, the slide was mounted in DPX.

### 4.14. SP11 Bioavailability Studies

#### 4.14.1. Chromatographic Conditions

As described [81], the separation and retention of analytes were performed on a Shiseido cap cell pack C18 column (S-5, 4.6 mm × 250 mm). The mobile phase for SP11 was composed of 2% acetic acid in HPLC grade water (A) and HPLC grade acetonitrile (B) (40:60, *v*/*v*). With 1 mL/min flow rate and 312 nm detection wavelength, 20 μL of samples were injected in columns maintained at room temperature. The mobile phase was degassed and filtered through a 0.45 μm membrane filter.

#### 4.14.2. Sample Preparation

Swiss albino mice were divided into two groups (n = 2) and SP11 was administered intraperitoneally to both groups. The mice in both groups were administered a single dose of SP11 at 50 mg/Kg b.wt and blood collected at different time points (5, 10, and 15 min, and 1, 3, 6, 12, and 24 h). As described [81], plasma samples were collected by centrifuging at 1500 rpm for 15 min. The analyte was then extracted from plasma using protein precipitation procedure and an equal volume of acetonitrile was added to the plasma. This was followed by a 2 min vortex; 14,000 rpm 5 min centrifuge and the upper organic layer was collected for analysis.

#### 4.14.3. HPLC Analysis

To evaluate linearity, a mouse plasma calibration curve was prepared with a gradient of SP11 (88% purity; 100, 200, 300, 400, and 500 μM) in the mobile phase. Selectivity was assessed by comparing the chromatograms of blank plasma and plasma spiked with known concentrations of SP11. LOQ and LOD for SP11 were calculated using the linear regression method.

### 4.15. Leukemia Xenograft Study

Nude mice (n = 14) were implanted with Molt4 (5 × 10^6^) cells. The mice were given 13 oral doses of either the vehicle control (control, n = 4) or SP11 (50 mg/kg; treated, n = 6) daily. Changes in the tumor size were observed for 26 days from the day of treatment. Tumor volume was calculated using the formula V = 0.5 × a × b^2^, where V is tumor volume, and a and b are major and minor tumor diameters.

### 4.16. Docking Studies

Protein structure 7L7J, a human HSP90 in closed state with p23 was selected in the docking study (at resolution 3.10 A, was the only entire Human HSP90 structure). The structure was cleaned for p23 and chain A was selected from the HSP90 dimer, further cleaned for heteroatoms and water molecules not belonging to the protein chain followed by energy minimization, performed in chimera keeping no atoms particularly as fixed. Hydrogen atoms were added to the structure also considering the H-bonds for minimization and protonation states for histidine were considered. Solvent AMBER ff1 4SB was used for the minimization to mimic the behavior of the protein in biological systems.

Autodock vina was used to dock the ligand SP11 with the HSP90 structure and LigPlot+ software for visualizing the protein ligand Interactions.

### 4.17. Cloning, Expression, and Purification of HSP90 C-Terminal Domain (CTD) and N-Terminal Domain (NTD)

To identify the differential interaction of the drug candidates with the CTD and NTD, both the domains had to be isolated. For this, HSP90 CTD (621 bp) and NTD (681 bp) were PCR amplified from MOLT-4 cDNA using the primer sequence given below.

HSP90 CTD

Forward primer: GGAATTCGAGTACTGTGTCCAACAG

Reverse primer: CCGCTCGAGGTCTACTTCTTCCATGCG

HSP90 NTD

Forward primer: GGAATTCGACCAACCGATGGAGGAG

Reverse primer: CCGCTCGAGAGCCTCATCATCGCTTACTTC

PCR cycle details are given in Table 2.

The products were then cloned between Xho1 and EcoR1 restriction sites in pET28a bacterial expression vector. Restriction digestion was performed using Xho1 and EcoR1 to confirm the insert release and clones were further confirmed using Sanger sequencing.

Protein was induced in BL21 cells. Overnight induction at 16 °C with an IPTG concentration of 0.25 mM was used for NTD and induction at 37 °C for 4 h at an IPTG concentration of 0.5 mM was used for CTD. Both the proteins were purified by Ni-NTA affinity chromatography using Profinity™ IMAC Ni-Charged Resin (BIO RAD). Purified protein was kept for dialysis overnight in 1X PBS. The protein concentration was determined by the Bradford method and the quality of the protein was analyzed using SDS-PAGE.

### 4.18. Isothermal Calorimetry

The binding affinity of drug molecules with HSP90 domains was evaluated through isothermal titration calorimetry (ITC) using Nano-ITC (TA Instruments, New Castle, DE, USA). The samples were degassed thoroughly before loading to remove air bubbles. Reference cell was filled with 300 µL 1X PBS and 300 µL of protein sample (10 µM) was loaded into the sample well. In the injection syringe, 50 µL of the compound was loaded. In a single experiment, 20 injections of 2.5 µL compound were titrated against the protein with 120 s injection intervals between consecutive titrations. Experiments were maintained at 25 °C with a stirring rate of 350 rpm. Since the drug candidate was dissolved in DMSO, a blank with only DMSO was also titrated against the protein.

### 4.19. Statistical Analysis

For statistical analyses, Excel 2010 software and Prism 5 software package (GraphPad, San Diego, CA, USA) were used. The values are expressed as mean ± SE in bar graphs, and one-way and two-way ANOVA were performed to compare differences between control samples and treatment groups. Values with *p*-value less than 0.05 are considered as significant and statistical significance is represented as **** (*p*-value ≤ 0.0001), *** (*p*-value ≤ 0.001), ** (*p*-value ≤ 0.01), * (*p*-value ≤ 0.05).

## 5. Conclusions

In summary, SP11 was synthesized, and its cytotoxicity was evaluated in leukemic cells and two independent lymphoma mouse models—allograft and xenograft. SP11 induced apoptosis rather than necrosis in cancer cells. SP11 exhibited minimal toxicity towards normal cells. Docking studies and isothermal calorimetry studies showed that SP11 binds to the C-terminal domain of HSP90. SP11 regulated a series of HSP90 client proteins both in vitro and in vivo. One of the most convincing aspects of SP11 administration was that it led to tumor regression with minimal drug toxicity and good bioavailability. SP11 thus has the potential to be developed as a novel anticancer agent.

## Figures and Tables

**Table 1:** IC_50_ values for 48 h SP11 treatment on leukemic cell lines.

Cell Line	IC_50_ (µM)
Molt4	0.72
Nalm6	0.847
CEM	0.889
Reh	1.06
K562	1.26

**Table 2:** PCR conditions for CTD and NTD of HSP90.

	CTD	NTD
Initial denaturation	95 °C, 5 min	95 °C, 5 min
Denaturation	95 °C, 30 s	95 °C, 30 s
Annealing temperature	50 °C, 35 s	56.1 °C, 35 s
Extension	72 °C, 40 s	72 °C, 40 s
Final extension	72 °C, 5 min	72 °C, 5 min
Total no. of cycles	34	34

## Data Availability

No new data were created or analyzed in this study. Data sharing is not applicable to this article.

## Supplementary Materials

The following supporting information can be downloaded at: https://www.mdpi.com/article/10.3390/molecules28135226/s1. Figure S1: Evaluation of Molt4 cell viability after SP11 treatment as tested by LDH assay. Data from 3 different biological replicates were collected for all experiments and were presented as bar graphs using Graph Pad prism tool. Two-way ANOVA followed by Tukey’s multiple comparison test was carried out and significance is represented as *** *p* value < 0.001, ** *p* value < 0.01, * *p* value ≤ 0.05, ns = nonsignificant. Figure S2: In Cell western(ICW) images for Bcl2 (A), Cleaved caspase-9 (B) and Bax (C) in Molt4 cells after SP11 treatment. (D) Background control image of ICW. Figure S3: (A) HSP90 CTD purification using Ni-NTA method. Wash 1,2 contains 50 mM imidazole and wash 3,4 contains 100 mM imidazole. Elusion 1, elusion 2 contains 400 mM imidazole and elusion 3 and 4 contains 1 M imidazole. (B) HSP90 NTD purification using Ni-NTA method. Wash 1 contains 20 mM imidazole and wash 2 contains 50 mM imidazole. Elusion 1 contains 200 mM imidazole and elusion 2 contains 500 mM imidazole. Figure S4: Thermodynamic binding profile for protein domain- ligand interaction.
