# Supplementary material for: A Coumarin–Imidazothiadiazole Derivative, SP11 Abrogates Tumor Growth by Targeting HSP90 and Its Client Proteins
Source: Molecules. 2023 Jul 5;28(13):5226. doi: 10.3390/molecules28135226 (PMC10343479; doi:10.3390/molecules28135226)

A Coumarin–Imidazothiadiazole Derivative, SP11 Abrogates Tumor Growth by Targeting HSP90 and Its Client Proteins

Snehal Nirgude [1,2,†], Shahana M. V. [1,†], Febina Ravindran [1], Sujeet Kumar [3,†], Shivangi Sharma [1,4,†], Raghunandan Mahadeva [1], Anisha Mhatre [1], Subhas S. Karki [3,*] and Bibha Choudhary [1,*]

1 Institute of Bioinformatics and Applied Biotechnology, Electronic City Phase 1, Bangalore 560100, Karnataka, India

2 Division of Human Genetics, Children's Hospital of Philadelphia, Philadelphia, PA 19104, USA

3 Dr. Prabhakar B. Kore Basic Science Research Laboratory Center (Off-Campus), Department of Pharmaceutical Chemistry, KLE College of Pharmacy, Rajajinagar, (A Constituent Unit of KLE Academy of Higher Education; Research, Belagavi), Bangalore 560010, Karnataka, India

4 Department of Biochemistry, Indian Institute of Science, Bangalore 560012, Karnataka, India

* Correspondence: subhasskarki@gmail.com (S.S.K.); vibha@ibab.ac.in (B.C.); Tel.: +91-080-233-256-11 (S.S.K.); +91-080-285-289-00 (B.C.); Fax: +91-080-234-253-73 (S.S.K.); +91-080-285-289-04 (B.C.)

† These authors contributed equally to this work.

Supplementary Figure S1: Evaluation of Molt4 cell viability after SP11 treatment as tested by LDH assay.Data from 3 different biological replicates were collected for all experiments and were presented as bar graphs using Graph Pad prism tool. Two-way ANOVA followed by Tukey's multiple comparison test was carried out and significance is represented as ***pvalue <0.001, **pvalue < 0.01, *pvalue <=0.05,ns=nonsignificant.

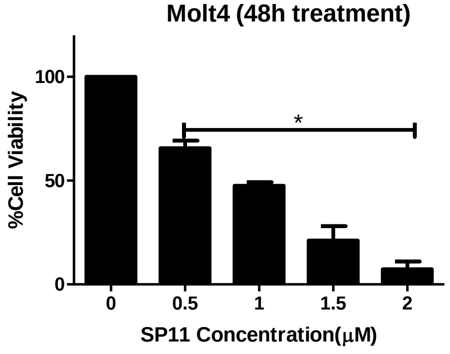

Supplementary Figure S2: In Cell western(ICW) images for Bcl2(A), Cleaved caspase-9(B) and Bax(C) in Molt4 cells after SP11 treatment. (D) Background control image of ICW.

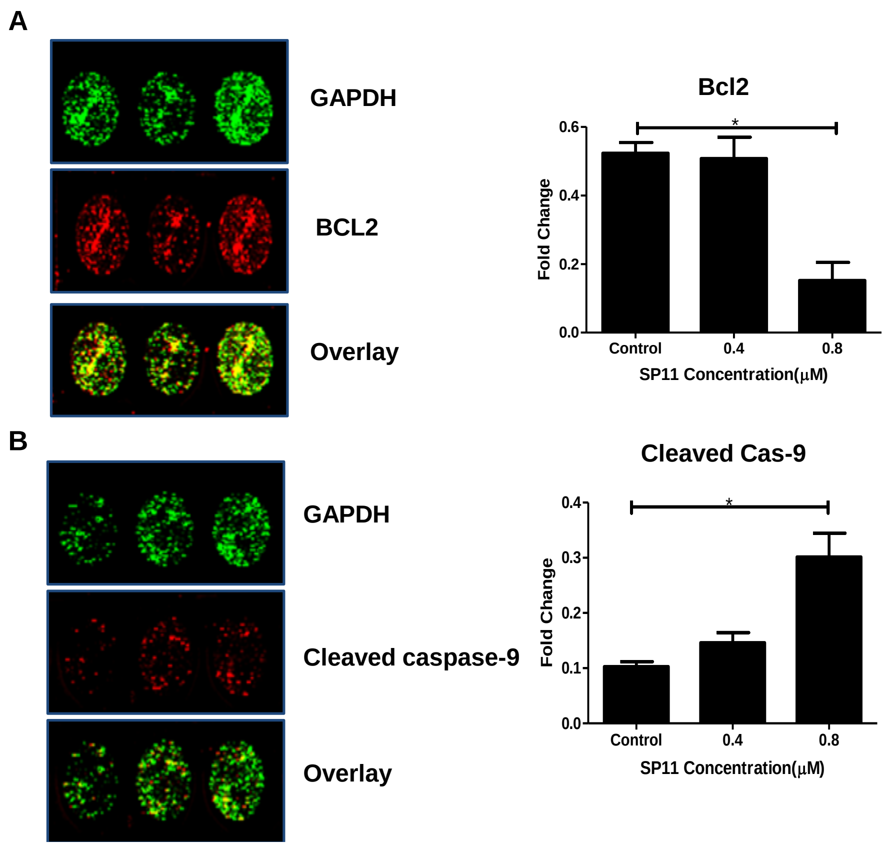
A
GAPDH
BCL2
Overlay
Bcl2
0.6
0.4
0.2
0.0
Fold Change
*
Control
0.4
0.8
SP11 Concentration(μM)
B
GAPDH
Cleaved caspase-9
Overlay
Cleaved Cas-9
0.4
0.3
0.2
0.1
0.0
Fold Change
*
Control
0.4
0.8
SP11 Concentration(μM)

C

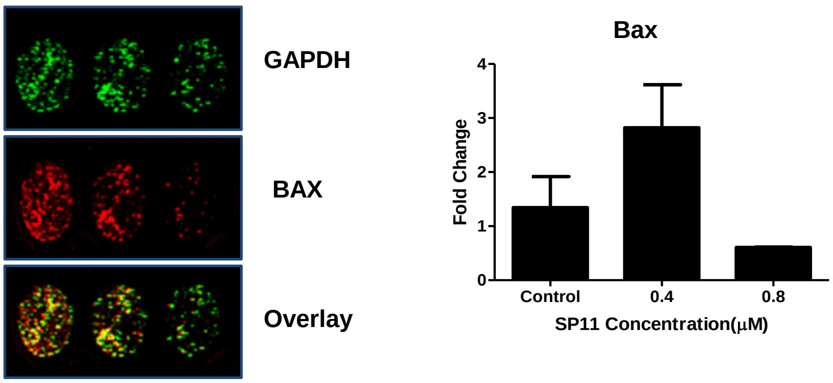

D

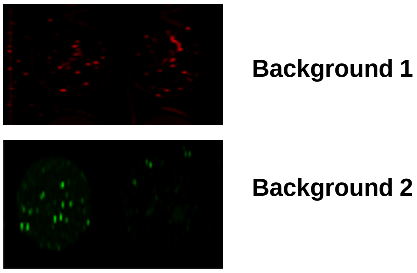

Supplemntary Figure S3: (A) HSP90 CTD purification using Ni-NTA method. Wash 1,2 contains 50mM imidazole and wash 3,4 contains 100mM imidazole. Elusion 1, elusion 2 contains 400mM imidazole and elusion 3 and 4 contains 1M imidazole.(B) HSP90 NTD purification using Ni-NTA method. Wash 1 contains 20mM imidazole and wash 2 contains 50mM imidazole. Elusion 1 contains 200mM imidazole and elusion 2 contains 500mM imidazole.

A

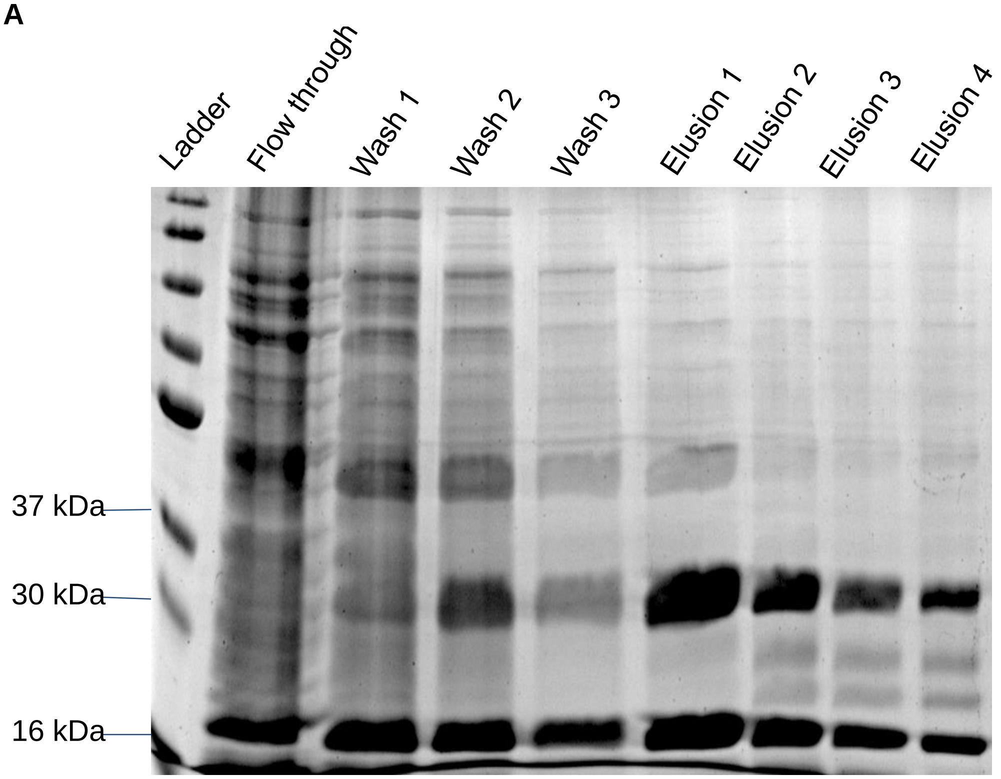
Ladder
Flow through
Wash 1
Wash 2
Wash 3
Elusion 1
Elusion 2
Elusion 3
Elusion 4
37 kDa
30 kDa
16 kDa

B

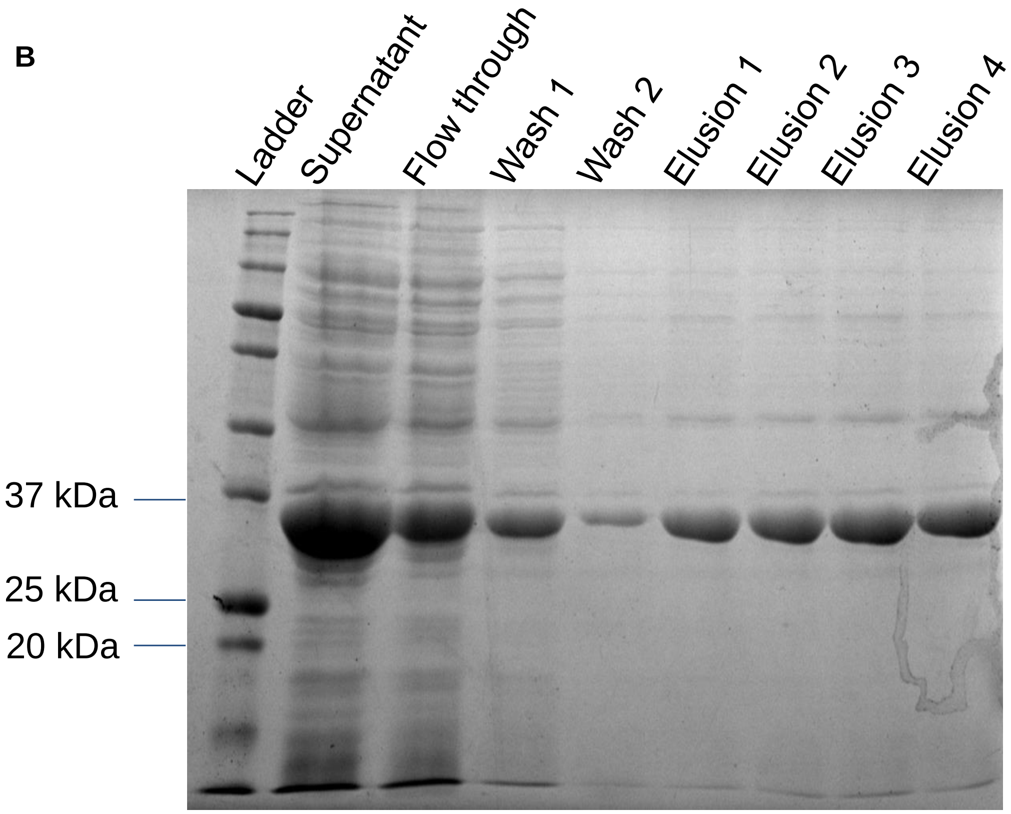
Ladder
Supernatant
Flow through
Wash 1
Wash 2
Elusion 1
Elusion 2
Elusion 3
Elusion 4
37 kDa
25 kDa
20 kDa

Supplementary Figure S4: Thermodynamic binding profile for protein domain- ligand interaction

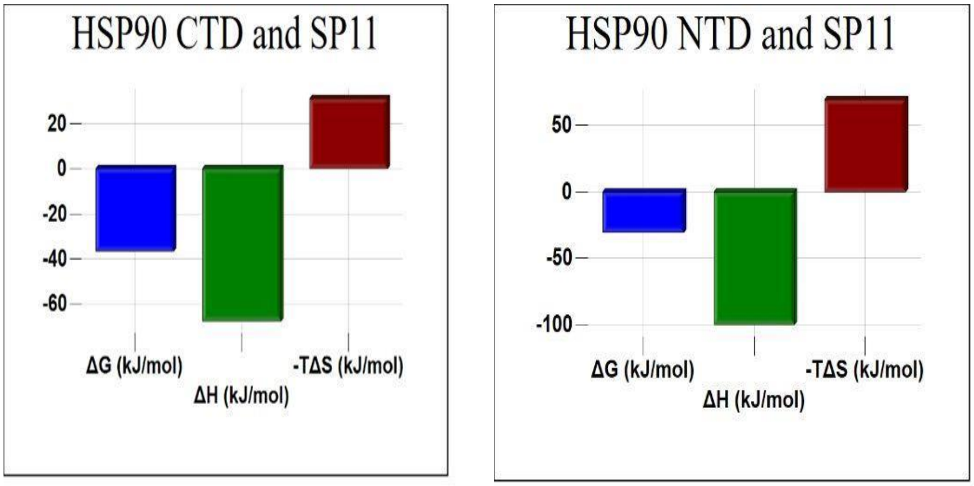

Supplement: Supplementary file 1 [file molecules-28-05226-s001.zip › molecules-2450396-supplementary.pdf]
